# The power of learning from the bottom up: working towards a blueprint for community-led biodiversity protection and restoration – ERRATUM

**DOI:** 10.1017/cft.2025.10007

**Published:** 2025-07-02

**Authors:** Emma Verling, Maria Power, Melanie Biausque, Lee Wah Pay, Aoife Deane, Rory Scarrott, Darragh Ó Súilleabháin

**Affiliations:** 1 MaREI, the SFI Research Centre for Energy, Climate and Marine Environmental Research Institute, University College Cork, Cork, Ireland; 2 Community Consultants, Waterford, Ireland; 3 British Geological Survey, Belfast, UK; 4 Cork County Council, Cork, Ireland

When this article was originally published in Cambridge Prisms: Coastal Futures the order of authors was incorrect. This has now been updated.

The publisher apologises for this error.
